# Fatty Acids and Cardiovascular Risk. Evidence, Lack of Evidence, and Diligence

**DOI:** 10.3390/nu12123782

**Published:** 2020-12-09

**Authors:** Francesco Visioli, Andrea Poli

**Affiliations:** 1 Department of Molecular Medicine, University of Padova, 35121 Padova, Italy; 2 IMDEA-Food, CEI UAM + CSIC, 28049 Madrid, Spain; 3 Nutrition Foundation of Italy, 20124 Milan, Italy; poli@nutrition-foundation.it

**Keywords:** fatty acids, cardiovascular disease, omega 3 fatty acids, omega 6 fatty acids

## Abstract

One of the most controversial areas of nutrition research relates to fats, particularly essential fatty acids, in the context of cardiovascular disease risk. A critical feature of dietary fatty acids is that they incorporate into the plasma membrane, modifying fluidity and key physiological functions. Importantly, they can reshape the bioavailability of eicosanoids and other lipid mediators, which direct cellular responses to external stimuli, such as inflammation and chronic stress conditions. This paper provides an overview of the most recent evidence, as well as historical controversies, linking fat consumption with human health and disease. We underscore current pitfalls in the area of fatty acid research and critically frame fatty acid intake in the larger context of diet and behavior. We conclude that fundamental research on fatty acids and lipids is appropriate in certain areas, but the rigor and reproducibility are lacking in others. The pros and cons are highlighted throughout the review, seeking to guide future research on the important area of nutrition, fat intake, and cardiovascular disease risk.

## 1. Introduction

Diet, as part of a healthy lifestyle, is one of the major determinants of human health. Even though the nutrition field is often plagued by over-hype and puffery [1], a consequence of the difficulties inherent to this kind of research, relevant data can be gathered from carefully performed epidemiological studies and clinical trials [2].

One of the most controversial areas of nutrition research is that of fats (particularly essential fatty acids), namely as related to cardiovascular disease risk. There are some major hurdles to overcome when studying the effects of fatty acids on the cardiovascular system and, particularly, on atherosclerosis and cardiovascular disease (CVD). One is that a large part of the available data should be interpreted under the overarching concept of “replacement” rather than direct effect [3]. An exception is that of omega 3 fatty acids when given in pharma-nutritional settings (see below) [4]. Another, and the probably more relevant, issue is that fatty acids are part of more complex food matrices, and, therefore, it is very difficult to single out the contribution of individual classes of these compounds to human health. In other words, the same fatty acid ingested as part of a plant-based diet versus an animal-based often appears to exert different effects [5,6,7], even it is difficult to explain such differences considering their obvious chemical identity.

One of the most important features of dietary fatty acids is that they become part of the plasma membrane, modifying its fluidity and, probably most important, reshape the availability of lipid mediators, such as the eicosanoids, in turn re-directing the cellular response to stimuli, such as inflammation.

It is important to underscore that most circulating and stored fatty acids are derived from the diet [8]. Hence, the extent and true nature of dietary fatty acids’ contribution to health and disease deserves in-depth analysis.

In this paper, we narratively review the most recent evidence linking fat consumption with health, we underscore current pitfalls of fatty acid research, and we critically frame fatty acid intake in the larger context of diet and behavior.

## 2. Dietary Fats: Why We Need Them?

Among the three macronutrients, i.e., carbohydrates, proteins, and fats, the latter, mainly composed of fatty acids part of triacylglycerols and, to a lesser extent, phospholipids, are relevant determinants of the health status [9]. Humans also ingest glycolipids, other complex lipids, and cholesterol. In many developing countries, fat intake is increasing, while in developed countries, fat intake has tended to decline over the past 30 years or so. Fats are quantitatively relevant in the diet because of at least two reasons: (1) easy availability due to massive cultivation of plants for oil production and (2) current low cost of energy-rich, fatty foods. We also need to eat fat for (a) energy provision; (b) essential fatty acid intake; (c) liposoluble vitamins’ intake; (d) augment food palatability (most flavors are liposoluble) [10]. In addition to the total amount of fat, it is important to note that the type of fat has also changed over time; the result is that the fatty acid composition of the human diet has changed. Data available from the UK indicate that the ratio of polyunsaturated to saturated fatty acids in the diet increased from 0.17 in 1959 to 0.22 in 1979 and to 0.40 in 1990 [11]. Much of this change has been brought about by a change in consumption habits, facilitated by public health bodies and scientific societies. Consumers moved from butter and animal fats to vegetable oils. In Europe, total fat intake ranges from 28.5 to 46.2% of total energy [12], of which polyunsaturated fatty acids represent 2.8 to 11.3%E [13].

In addition to providing the most quantitatively dense amount of energy, fatty acids play very relevant physiological roles [10,14]. Notably, selected types of fatty acids, such as the long-chain, highly unsaturated fatty acids of the omega 6 and omega 3 series, are important modulators of cell function and precursors of lipid mediators. In other words, dietary fat is indispensable for energy, delivery of lipid-soluble vitamins, and physiology. Even though the lay public (and physicians as well) often think that diets should be as low in fat as possible, the results of the largest ever randomized intervention trial, i.e., the Women Health Initiative (WHI) [15], have shown that limiting the intake of total fat does not significantly improve the cardiovascular risk profile, the incidence of type 2 diabetes and, probably, does not induce remarkable weight loss. Indeed, no significant protective effect of low-fat diets on cardiovascular end-points was observed in this trial, which comprised women who reduced their total fat intake from 35/37% to 25/27% for an average of 7 years, as compared to women who did not change their diet [15]. Recent epidemiological studies fully support this concept [16,17,18]. In the observational follow-up of the PREDIMED initiative [18], persons consuming about 50% of calories from fat had a large, i.e., almost 50%, reduction of the risk of all-cause mortality as compared to persons who derive about 30% of calories from fat, as most of the international guidelines suggest [19].

In summary, in Western countries, more emphasis should be placed on the relative proportion of individual fatty acids rather than on the overall reduction of fat intake.

### 2.1. Saturated Fat

The dietary intake of saturated fatty acids is an important determinant of plasma LDL cholesterol levels. Hence, this class of fatty acids has long been considered as positively associated with cardiovascular risk (the similar effect on plasma HDL cholesterol levels being usually overlooked). The debate is still going on, but the consensus that saturated fat consumption must be limited [20] is losing momentum [21]. At least two meta-analyses, in fact, reported no association between saturated fat intake and overall mortality [22,23]. The critical issue is that an unrestricted reduction of saturated fats leads to the ban of otherwise healthful food, such as dairy foods [22]. Additionally, the recent paper from the Global Burden of Disease 2019 group does not include saturated fats among the first 15 most important dietary determinants of death [24].

As discussed below, milk and derivatives are rich in saturated fatty acids (they account for about 41% of the total saturated fatty acids intake in the Holland EPIC cohort) [25] and contain ruminant *trans* fatty acids, whose consumption, in light of the data available to date, should not be discouraged [21,26]. Indeed, in addition to saturated fatty acids, milk also contains calcium and/or tripeptides with a mild ACE-inhibitory action, which might, in fact, induce a reduction of blood pressure [27] and, therefore, of the risk of cerebrovascular events, with varied effects on the risk of coronary events [26,28]. Further, milk consumption is being associated with lower coronary artery calcification progression [29], possibly because of its specific short-chain saturated fatty acids pattern [25]. In summary, milk is a complex food item [30], and those who eliminate it and its derivatives from their diet (generally due to real or presumed intolerance) might statistically experience a significant increase in the risk of developing diabetes or hypertension (one new case for every 15 subjects who stop using milk) [31]. Cheese intake, in a number of observational studies, is associated with a reduction of CVD events and stroke [26]. It is also of interest that the circulating or adipose tissue concentrations of the odd-chain fatty acids 15:0 and 17:0, which can be considered as a specific marker of intake of milk and dairy products (these fatty acids are not contained in significant amounts in other foods and cannot be synthesized by humans), are often associated, in observational studies, with a decrease of CVD events [32,33].

In the context of a varied and balanced diet, the consumption of saturated fat via milk (and probably of dairy products) does not, therefore, significantly influence cardiovascular risk [26]. Some comparisons between reduced fat and full fat dairy, moreover, suggest that the latter may be more effective in maintaining human health, namely cardiovascular health [34,35].

Another meaningful source of saturated fat is meat, namely that of ruminant origin. Because of this, its consumption is often associated with increased cardiovascular disease risk [36]. This chiefly applies to processed meat [37], and the consumption levels of 0.5 servings per day or less are not associated with increased cardiovascular risk [38]. For unexplored reasons, these associations are much less pronounced in European studies [39]. The increasing evidence that compounds other than saturated fatty acids (namely, carnitine and choline), which can be converted in TMAO by microbiota and liver FMO enzymes, may be responsible for the atherogenic effects of meat [40] also indirectly supports the idea that the role of saturated fatty acids, in this context, may actually be quite limited.

Interestingly, in the MESA study, saturated fatty acids from meat were associated with an increased incidence of cardiovascular disease, while saturated fatty acids from dairy showed an opposite association with the same risk [41]. This suggests that the food matrix may play a larger role than saturated fatty acids on these clinical endpoints.

The substitution of saturated fatty acids in the diet with other nutrients, according to available evidence, may yield different results: the substitution of polyunsaturated and monounsaturated fats (but not that of total carbohydrates, refined complex carbohydrates, or sugars) for saturated fats was associated, in two large US cohorts, with a reduction of CHD risk [42]. It should be considered that such observation could be attributed to the possible beneficial effect of polyunsaturated and monounsaturated fatty acids and not necessarily to an adverse effect of saturated fatty acids (SFAs) [36].

In summary, the uncritical advice to reduce intake of SFAs without considering specific fatty acids and food sources is not aligned with the current evidence base [36]. The view that saturated fat (a highly heterogeneous class of fatty acids) per se increases cardiovascular mortality is over-simplistic and distracts from the “food matrix” concept. Various saturated fatty acids have distinct metabolic roles [43], and much additional research is needed to untangle their relative roles in cardiovascular physiopathology [44].

### 2.2. Monounsaturated Fatty Acids (MUFAs)

International guidelines now promote the substitution of MUFA or polyunsaturated fatty acids (PUFA) for SFA. In view of the fading view of SFA as being deleterious (vide infra), such advice should be based on sound evidence of direct protective human effects of these fatty acids. As far as MUFAs are concerned, some evidence supporting this notion can actually be found in the published literature. Data from Crete (a region where longevity is amongst the highest in the world) show that calorie intake from fat approximates 40% of total calories, about half of which has been from olive oil, i.e., high intake of the MUFA oleic acid (18:1ω9) [45]. Similar effects have been observed in the PREDIMED trial [18].

Mostly based on the indisputable association between olive oil use and lower CHD incidence (as well as that of neurodegeneration and cancer [46]), many researchers and public health bodies attribute MUFAs direct healthful properties. However, a close look at the published literature casts some doubt on this assumption, and the effects of extra virgin olive oil in relation to coronary risk are to be attributed to its (poly)phenols and not to its lipid components such as oleic acid [46]. To begin with, several studies, summarized in highly-cited equations [47,48,49,50], calculated that the effects of MUFAs on cholesterol—when they isocalorically replace carbohydrates—are rather small or negligible. Further, a recent review by Tomé-Carneiro et al. [51] highlighted the potentially harmful effects of olive oil and oleic acid when used in excess.

Examples of the noxious effects of excessive MUFAs use include observational studies. In a cross-sectional study, relations between fatty acids intake and metabolic syndrome (MetS) status among overweight and obese women were addressed. Overweight women with MetS consumed higher amounts of monounsaturated fatty acids, polyunsaturated fatty acids, and linoleic acid compared to overweight women without MetS [52]. Further, in a large sample of Swedish middle-aged men (total 2322), a follow-up of 32 years showed that raised amounts of palmitic, palmitoleic, and oleic acid were related to a higher risk for stroke/transient ischemic attack, whereas a higher proportion of linoleic acid was protective [53].

Several case-control studies that analyzed fatty acids profiles in the circulation actually reported higher rather than lower CHD risk as associated with higher proportions of MUFAs. Examples include Block et al. [54], who reported higher oleic acid in the blood cell membranes of 768 acute coronary syndrome cases as compared with the same number of controls. These conclusions were reinforced by Marangoni et al. [55], who compared 119 patients with a recent diagnosis of myocardial infarction with 103 controls. Whole blood oleic acid concentrations were higher in the former than in the latter. Finally, a metabolomic study by Würtz et al. [56] classified circulating monounsaturated fatty acids as predictors of cardiovascular risk in 14,629 participants to three population-based studies carried out in Finland and the UK. Of note, recent research indicates that the effects of plant-derived monounsaturates are different than those found in meat [57]. These interesting data, which need to be confirmed in larger settings, point to a larger relevance of the food matrix than the fatty acid composition.

While the near totality of dietary MUFAs is oleic acid, it is worth devoting a brief discussion to the role of palmitoleic acid (16:1ω 7): its dietary intake is quite low (main sources are macadamia and sea buckthorn oils, neither of which is consumed in quantities worth noting worldwide), and this fatty acid is considered as a marker of lipogenesis, particularly important in cardiometabolism, namely in the etiology of non-alcoholic fatty liver disease (NAFLD). Among the proposed activities of omega 3 fatty acids (see below), their inhibition of hepatic lipogenesis via inhibition of the LXR-SREBP-1c system [58] might be an often-overlooked healthful one [59,60,61,62].

### 2.3. Polyunsaturated Fatty Acids (PUFA)

The main PUFA in the diet is linoleic acid (18:2ω6, LA), followed by alpha-linolenic acid (18:3ω3, ALA). Their intakes among adults in the Western world are approximately 13.5 g and 1.7 g per day, respectively. Longer-chain PUFAs are consumed in lower amounts than LA and ALA. Arachidonic acid (20:4ω6, ARA) intake in Western populations fluctuates between 50 and 300 mg/day. It is important to underscore that, although several fatty acids can be synthesized in the human body, mammals are able to insert double bonds in fatty acids’ position 9 but are unable to do so in positions 3 and 6, which is indispensable to synthesize PUFAs [43]. Consequently, linoleic and alpha-linolenic acids need to be ingested with the diet and are, therefore, termed “essential”. Conversely, longer chain ω6 and ω3 can be obtained, even if with different efficiency, from the aforementioned 18 carbon PUFAs, through the intervention of specific desaturases (∆5 and ∆6) and elongases.

On the other hand, fatty acid essentiality might also refer to the several indispensable roles they play in cellular physiology.

It is common wisdom that the Western diet, if often too rich in fat, mainly as saturated, monounsaturated, and polyunsaturated fatty acids of the omega 6 series (such as LA), present in high concentrations in most seed oils [63]. It has been suggested that this theoretical unbalance leads to a “dilution” of omega 3 fatty acids, usually scant in most common foods [64]. However, this notion is being disproven by accumulated evidence. At least in Europe, consumption of omega 6 PUFAs is actually below the levels recommended by several scientific bodies, such as the American Heart Association (see below).

### 2.4. Omega 6 Fatty Acids

As mentioned, the most abundant omega 6 PUFA in the diet is LA (18:2ω6), whose intakes are, on average, 50-fold higher than those of ARA [65,66,67]. Research on LA gained traction approximately 50 years ago [68] when this fatty acid was shown to decrease blood cholesterol concentrations. Consequently, dietary advice to increase its consumption was issued by public health authorities. Indeed, polyunsaturated fat (again, primarily LA) in the US increased from approximately 3% of energy in the 1950s to about 6–7% of energy [69,70]. The increase in LA consumption was associated with a marked (50%) reduction of cardiovascular disease in the US [69]. This profound dietary change was confirmed by large increases in the LA acid content of adipose samples. The effects of omega 6 PUFAs (and of other fatty acids) on blood lipids have been calculated by equations that yielded almost identical results: consumption of saturated fat is positively related, and polyunsaturated fat is inversely related to serum cholesterol concentrations [47,49]. These findings were corroborated by Mensink et al. [50], who also addressed the effects of dietary fats on not only total blood cholesterol but also LDL and HDL cholesterol and triglyceride levels [50]. In summary, compared to carbohydrates, polyunsaturated fat (the vast majority of which is, as mentioned above, omega 6 PUFA) reduces LDL cholesterol, with little effect on HDL cholesterol and triglycerides. Hence, dietary intake of omega 6 fatty acids, e.g., the substitution of 5% of calories from saturates with linoleic acid, was associated with a significant reduction in cardiovascular risk (−9%) in a US meta-analysis [71]. The association between increasing LA intake and reduction in CHD events holds, in this meta-analysis, up to at least 8% of total calories from LA. Two recent meta-analyses have shown a negative association of LA plasma or tissue levels (which reflect the intake of this essential fatty acid) with CHD and CVD incidence and mortality [72] and type 2 diabetes incidence [73].

Mechanistically, it has been suggested that LA may partially inhibit the enzyme PCSK9, which regulates the hepatic LDL receptor’s degradation, in a manner similar to (albeit much less effective than) that of currently available drugs, i.e., alirocumab and evolocumab. This hypothesis, however, requires further confirmation. Further, plasma levels of linoleic acid positively correlate with insulin sensitivity [71]. The net result of using recommended amounts of omega 6 fatty acids is that of cardiovascular risk reduction. Aside from epidemiological studies, one arm of the clinical trial PREDIMED, i.e., the one where patients were given linoleic acid-rich dried fruit and nuts, confirmed cardiovascular protection [74].

Theoretically, omega 6 fatty acids, namely arachidonic acid, could be pro-inflammatory, being the precursors of eicosanoids. From a nutritional viewpoint, some theories posit the competitive relationship with omega 3 fatty acids, with which omega 6 should compete for some enzymatic activities, i.e., elongation and desaturation [75]. However, the majority of human studies do not confirm this biochemical hypothesis [69], and a systematic review of the literature did not actually conclude on any increase in inflammatory markers associated with the consumption of omega 6 in humans [76]. Of note, a case-control study of Italian-infarcted subjects reported that high plasma levels of both omega 3 and omega 6 were associated with a clear risk reduction, while the now dismissed [77] omega 6/omega 3 ratio did not correlate significantly with the risk itself [55]. Additionally, the omega 6/omega 3 ratio was negatively associated with the overall death rate, i.e., increasing ratios were associated with decreasing the risk of death for any cause in a recent and very large prospective cohort [78].

Even though it is difficult to study the direct effects of ARA in humans, via RCTs, a review of dietary surveys of ARA intake indicates that the amount of ARA intake is 100–250 mg/day in advanced counties. ARA supplementation (82 or 120 mg/day for 3–4 weeks) at a dose equal to or less than the dietary ARA intake increases plasma ARA composition; plasma ARA composition is ARA dose-dependently increased in the range of 82–3600 mg/day; and ARA supplementation decreases plasma LA composition but not docosahexaenoic/eicosapentaenoic acids composition [67]. To date, nine RCTs of ARA have been performed, and none of them reported increases in inflammation markers [67]. Conceivably, there is a large array of lipid mediators that originate from ARA, not limited to inflammatory eicosanoids, and the sum of their biological activities lead to null effects on inflammation [79]. In conclusion, neither LA nor ARA intakes are associated with increased inflammation [76]. On the contrary, there is evidence of synergistic anti-inflammatory actions of LA and omega 3 fatty acids (see below) [80], leading to a 54% lower risk of total mortality (HR, 0.46; 95% CI, 0.30–0.69) relative to those with lowest levels of both [80].

Finally, contrary to popular belief, the intake of omega 6 fatty acids is quite low in Europe and in the USA [12,13]. According to an INRAN-SCAI survey, in which omega 6s were unfortunately reported together with omega 3 polyunsaturates, the cumulative PUFAs intake in Italy covers only 4–5% of total calories [81]. In conclusion, an increase in omega 6 dietary intake from seed oils, vegetables, and nuts could help to further reduce coronary risk, especially in countries where consumption is sub-optimal [70].

### 2.5. Omega 3 Fatty Acids

In physiological terms, omega 3 PUFAs, especially those which are most relevant in biological terms, i.e., the long-chain PUFAs eicosapentaenoic (EPA) and docosahexaenoic (DHA), have become analogous to micronutrients (intakes of few hundreds mg/day in most populations out of approximately 100 g/day of total fat). Irrefutable evidence shows that an adequate dietary intake of omega 3 from plant foods (linseed, canola, and soybean oils and walnuts), namely alpha-linolenic acid (ALA; 18:3ω3), and from fish (EPA and DHA, respectively) is associated with a significant reduction of coronary risk and sudden death [82,83], especially in the elderly [84]. Dimensionally, data from 17 prospective cohort studies examining the association of dietary EPA and DHA with the risk of various coronary outcomes indicate an 18% risk reduction for any CVD event for those with higher dietary intake of EPA + DHA compared to those with lower intake [85]. Significant reductions of 23%, 19%, and 47% in the risk for fatal coronary events, coronary death, and sudden cardiac death, respectively, were also computed [85]. These protective actions, which come from observational studies and are mostly associated with fish consumption rather than supplement use, are mechanistically explained by the favorable effects of EPA and DHA on triglyceridemia, on platelet function, blood pressure, and on the lower production of adhesion and proinflammatory proteins by the arterial wall [82], in addition to direct antiarrhythmic and antioxidant effects [82,86,87]. The first RCT that investigated the pharmacological use of omega 3 was the GISSI-Prevenzione, in which capsules of omega 3 ethyl esters concentrates (at a dose of 850 mg/day) vs. placebo were administered to subjects with a clinical history of a coronary event. This trial clinically confirmed the cardioprotective effects of EPA and DHA [88,89]. The subsequent JELIS RCT studied the use of EPA (1.8 g/d as an ethylester) alone in Japanese patients treated with statins and reported a 19% lower incidence of reinfarction compared with the statin alone group. After these studies, however, no further significant evidence emerged, from controlled trials, in favor of treatment with omega 3 in secondary prevention, with the exception of the REDUCE-IT trial, where 4 g/d of icosapent ethyl significantly reduced cardiovascular events in hypertriglyceridemic, statin-treated patients. In terms of lipid profile, there was a significant reduction in triglycerides (a decrease of 0.5 mmol/L; *p* < 0.001) and LDL-cholesterol (a decrease of 0.13 mmol/L; *p* < 0.001). Of note, the numbers needed to treat computed from the Bhatt et al. study is 70 [90]. Consequently, on November 14, 2019, the USA Food and Drug Administration approved the use of icosapent ethyl (registered trademark: Vascepa^®^) as an adjunct to statin therapy in patients with elevated triglyceride levels.

Other trials have been performed with high (higher than the previous ones) doses of pure EPA. In this respect, the small, i.e., 193 patients, and non-blinded Japanese CHERRY (combination therapy of eicosapentaenoic acid and pitavastatin (PTV) for coronary plaque regression evaluated by integrated backscatter intravascular ultrasonography) study, which used intravascular ultrasound, reported that combination EPA (1.800 gr/d)/PTV therapy significantly reduced coronary plaque volume compared to PTV therapy alone [91].

Very recently, Lazáro et al. [92] investigated whether serum-PC EPA (proxy for marine omega 3 consumption) levels at the time of ST-segment elevation myocardial infarction (STEMI) were associated with a lower incidence of major adverse cardiovascular events, all-cause mortality, and readmission for cardiovascular (CV) causes at 3 years’ follow-up. Elevated serum-PC EPA and ALA levels at the time of STEMI were associated with a lower risk of clinical adverse events, which the authors attribute to diets rich in those two fatty acids [92]. A recent sub-analysis of the ANCHOR study [93] reported that icosapent ethyl 4 g/day significantly and safely reduced triacyclglycerols (TG) and other atherogenic and inflammatory parameters without increasing LDL-C in statin-treated patients with hsCRP ≥ 2.0 mg/L and TG 200 to 499 mg/dL at baseline, versus placebo [93].

The rationale for using pure EPA instead of the more habitual fish oil mix of EPA and DHA is that the latter purportedly increases LDL-cholesterol concentrations more than the former [94,95,96], at least in hypertriglyceridemic patients [97]. This effect is more pronounced in ApoE4 carriers and may, in part, negate the cardioprotective action of DHA in this population subgroup [98].

Even though the VITAL trial performed by Manson et al. recently concluded that “supplementation with n-3 fatty acids did not result in a lower incidence of major cardiovascular events or cancer than placebo” in 25,871 participants who underwent randomization in a two-by-two factorial design trial of vitamin D_3_ (at a dose of 2000 IU per day) and marine n-3 fatty acids (at a dose of 1 g per day) in the primary prevention of cardiovascular disease, a close inspection of the tables shows that the hazard ratios for total myocardial infarction were 0.72 (95% CI, 0.59 to 0.90), those for death from myocardial infarction were 0.50 (0.26–0.57), and those for percutaneous coronary intervention were 0.78 (0.73–0.95), i.e., statistically significant [99]. A more updated meta-analysis by the same senior author concluded that marine omega 3 supplementation lowers the risk for myocardial infarction, CHD death, total CHD, CVD death, and total CVD, even after exclusion of REDUCE-IT [100]. Risk reductions appeared to be linearly related to marine omega 3 dose [100].

The ongoing EVAPORATE trial [101], where 4 g/d of pure EPA was administered to cardiovascular patients with triglyceride concentrations 200 to 499 mg/dL, just reported significant regression of low-attenuation plaque volume measured by serial multidetector computed tomography [90,102] and helped shed some light on the dose issue [103].

On the other hand, the recent STRENGTH (Statin Residual Risk Reduction with Epanova in High CV Risk Patients with Hypertriglyceridemia) trial, evaluating the effect of Epanova^®^ 4 g daily compared to placebo (corn oil) on reducing the risk of major adverse cardiovascular events in 13,086 patients on optimal statin therapy with mixed dyslipidemia and at high risk for CV disease, was stopped early due to its low likelihood of demonstrating a benefit [104]. Finally, an RCT of krill oil, i.e., omega 3 mostly as phospholipids, carried out in 242 patients with severe hypertriglyceridemia, was also stopped after 12 of the programmed 46 weeks because of a strong placebo effect and the very recent Bischoff et al. publication reported no significant differences in improvement in systolic and diastolic blood pressure of older adults given 1 g/d of algal oil (330 mg of EPA and 660 mg DHA) [105]. Finally, a very recent trial of 930 mg/d EPA and 660 mg/d DHA vs. corn oil given to elderly patients failed to prove benefits [106]. In synthesis, the current clinical evidence of cardioprotective effects of EPA and DHA therapy is quite sparse [107] and not strong enough for public health bodies to incorporate them in the therapeutic toolbox of myocardial infarction patients. However, some data are suggestive of salutary actions, which should be further investigated (see below).

It is important to underline that the largely negative outcomes of the most recent, i.e., since 2007, trials should not undermine the recommendation to regularly consume fish or plant sources of ALA [108,109]. Several reasons may, in fact, explain the lack of effect reported by the most recent trials of supplementary fish oil [110,111], notably: (a) as opposed to the early trials, such as the GISSI-Prevenzione, cardiopathic patients currently receive highly effective multi-drug pharmacological treatments; the effect of adding omega 3 fatty acids “on top” of these treatments is likely very small; (b) the fatty acid profiles of RCT patients have been almost never evaluated: some subjects could benefit more than others from treatment with omega 3 (for example, those with low basal blood levels of these fatty acids) [103]. From a mere pharmacological viewpoint, this is surprising because, for instance, the Omega 3 Index (%EPA and DHA in erythrocytes, measured with a standardized methodology) is a risk predictor for cardiovascular mortality (CVD). Namely, CVD is 30% lower with an omega 3 index >8% as compared with an omega 3 index of <4% [112]. As recently reported by Bittner et al. [113], a low Omega 3 Index is associated with early-onset coronary atherosclerosis. Ten cohort studies identified a 15% reduction in risk of fatal CHD for each one standard deviation increase in omega 3 index [112]. In many trials, omega 3 capsules might have been given to people who did not necessarily need them, and too low of a dose might have administered to people with a very low Omega 3 Index. This resembles running hypertension trials without measuring blood pressure or hyperlipidemia trials without measuring LDL [114]; (3) the bioavailability of omega 3 fatty acids administered as ethylesters, as opposed to, e.g., triacylglycerols and phospholipids from fish, is questionable. As reviewed by Schuchardt and Hahn [115], bioavailability heavily depends on the concomitant intake of fat and/or adequate volume of foods, has high inter-individual variability, and so forth. Examples of good bioavailability include omega 3 fatty acids formulated into milk products [116], when eaten with salmon vs. capsules [117,118], or when administered as krill oil [119]—none used in the trials meta-analyzed. Another often overlooked and controversial [120] issue is that fish oil is, by its own nature and be it from diet [121] or supplements [122,123], prone to oxidation [124]. Physicians should advise patients to store their medications/supplements in the dark and even freeze fish oil capsules to avoid the formation of noxious [125] peroxides.

In summary, bi-weekly consumption of fish (especially fatty fish) remains a cornerstone of cardiovascular prevention and should provide an average of 500 mg/d of EPA and DHA [126,127]. The use of EPA and DHA in secondary prevention does not currently rest on solid ground because of the aforementioned reasons; yet, given the scantiness of side effects, some patients could benefit from additional fish oil intake, and physicians should discuss its use with them [128].

Interest in ALA gained momentum at an International workshop, whose conclusions were published in the year 2000 by Crawford et al. [129]. Due to its inefficient conversion to EPA and DHA [130], ALA has been often neglected as bioactive omega 3 fatty acid. The only secondary prevention trial in which ALA has been employed (as an ingredient of margarine) was the Lyon Heart Study [131], which was interrupted early because of statistically significant results, had a small number of events, and has never been replicated. Its follow-up, however, confirmed the positive effects of ALA on reinfarction rates [132]. Another small crossover Japanese trial recently reported enhanced fat oxidation in healthy subjects, administered with 2.5 g/d of ALA-enriched diacylglycerol vs. placebo [133], suggesting its use in visceral obesity prevention. Other evidence indicative of the cardioprotective effects of ALA come from epidemiological data linking high consumption of ALA-rich foods, such as walnuts [134,135], flaxseed [136], and some seed oils [137]. Currently, large-scale primary or secondary prevention trials are missing, and, in summary, the cardiovascular actions of ALA have been poorly explored and remain to be fully elucidated [109].

### 2.6. Trans Fatty Acids

*Trans* fatty acids are unsaturated fatty acids with at least one double bond in the *trans* configuration, which results in a straighter molecular shape than with a double bond in a *cis* configuration. *Trans* fatty acids are not essential, and they do not serve any vital functions [138]. Conversely, the consumption of *trans* fatty acids (usually identified on food labels as “partially hydrogenated vegetable fats”) has untoward effects on lipid and lipoprotein metabolism, i.e., increase in LDL cholesterol, reduction of HDL cholesterol, and deterioration of endothelial function, perhaps due to their pro-inflammatory actions. There is a clear direct association between consumption of *trans* fatty acids with food and coronary risk [23]. Indeed, even though correlation does not mean causation, US counties where the intake of *trans* fatty acids has been banned are witnessing a decrease in cardiovascular events [139].

Recent analyses of erythrocytes from European subjects indicate that levels of industrial *trans* fatty acids appear to be decreasing, as are those of ruminant-derived C16:1n-7t, a marker for dairy and meat intake [140]. Altogether, the average intakes in European countries of ~1 energy percent [141] and less than that in the United Kingdom, with a major proportion coming from dairy products and meat [142]. In France, for example, *trans* fatty acids’ intake is now exclusively from ruminant meat and dairy products [143]. The USA is also witnessing a slow reduction in industrial *trans* fatty acids use. In Europe and the USA, the average intake of *trans* fatty acids from ruminant sources is around 0.5 energy percent [138].

One matter worth discussing is the conventional classification of *trans* fatty acids in two groups: artificial *trans* fatty acids (of industrial origin) and natural *trans* fatty acids (produced by ruminants) [138]. This leads to a discussion of whether the latter have cardiovascular actions different than the former. Indeed, milk fat naturally contains ~5% *trans* fatty acids, resulting in very low intakes. Few observational studies have investigated separate effects of dietary ruminant *trans* fatty acids, which ranges from 2.8–10 g/day [144]. No adverse effects of ruminant *trans* fatty acid intake were noted [144], in line with previous conclusions [23]. On the contrary, some authors propose putative effects of ruminant *trans* fatty acids [145,146,147]. Of course, those fatty acids are part of more complex food items, and it is difficult or even impossible to single out their true contribution to human health and pathology.

In summary, the ban on the consumption of *trans* unsaturated fatty acids, typical of old margarine used in dough and some low-quality baked goods, should be implemented. The roles played by ruminant trans fatty acids in human health remain to be carefully studied via appropriate trials.

## 3. Conclusions

The fatty acids and cardiovascular disease field (in addition to other ones, such as cognitive decline [148,149]) is a rapidly evolving one, mainly due to the difficulty of performing appropriate clinical trials where fatty acids replace carbohydrates rather than one another. However, some firm conclusions can be drawn. The first one concerns SFAs that are highly heterogeneous in nature and whose effects most likely depend on the food matrix and on the other classes of fatty acids they substitute for in the diet. International scientific societies recommend keeping their intake below 10%E, but more attention should be probably paid to their source rather than their absolute amount.

MUFAs, apparently, play minor roles in the CHD and CVD scenario but, when ingested via extra virgin olive oil, convey (poly)phenols with interesting cardioprotective properties.

Omega 6 PUFAs became very popular in the late 70s when Dr. Ancel Keys and coworkers undertook the Seven Countries study [150], whose results prematurely [151] advocated the use of seed oils rather than butter. Then, the theory that predicts that the essential omega 6 fatty acids, namely linoleic acid, increase inflammation because they are precursors of eicosanoids led some investigators to classify them as harmful. As reviewed above, this theory proved unsound in humans, and consumption data actually indicate that linoleic acid use is often below recommended levels. Hence, there is—at present—no sound evidence to suggest that they should be looked upon as harmful, and there is no reason to worry about the proportion of calories they provide within a healthy diet.

Concerning omega 3 fatty acids and cardiovascular disease, the state-of-the-art is as follows: enhanced intakes of omega 3 fatty acids are cardioprotective via modulation of various parameters. The effects are obtained with relatively low amounts of omega 3, in the order of ~500 mg–1 g/day, which is a tiny fraction of total fat ingested daily (around 100 g in most countries [12,13]). One interesting trait of omega 3 fatty acids is, indeed, that their accrual in plasma and cell lipids is relevant, even at such relatively low levels of intake. Further, EPA and DHA mainly accumulate in rather stable lipid pools where they are retained even after long washout periods [152]. When employed in cardiovascular therapy, the effects of DHA and, particularly, EPA appear at much higher doses than the dietary ones. The current impasse of fish oil use in cardiovascular secondary prevention requires a redirection of our approach to the pharmacology of these molecules, which should chiefly implicate reflections on doses and formulations. The advice to consume omega 3-rich oily fish, nuts, and leafy vegetables rests on solid evidence. Current research on transgenic plants that provide EPA and DHA [153] might evolve into the formulation and marketing of novel food items.

**Figure 1:**
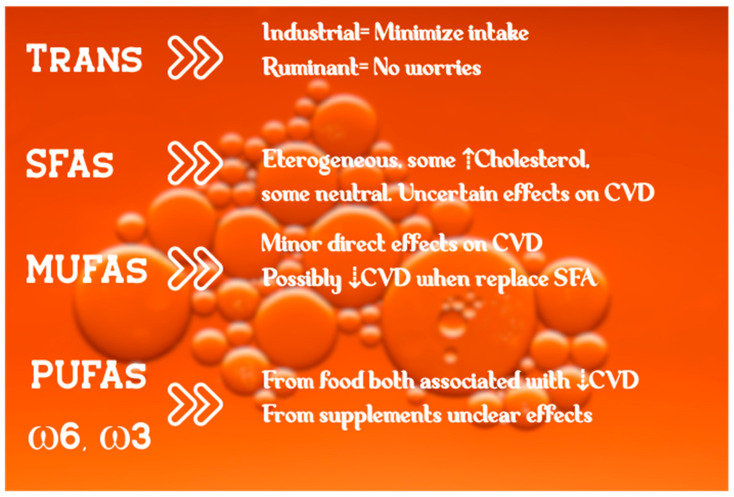
Schematic view of fatty acids’ effects on the cardiovascular system. Photo by Sharon Pittaway on Unsplash.

In conclusion, some evidence on the effects of fatty acids and lipids solidified over the years, thanks to proper research (Figure 1) [154]. Other issues need to be elucidated and will be the subject of future investigation.
